# Aspen pectate lyase *Ptxt*PL1-27 mobilizes matrix polysaccharides from woody tissues and improves saccharification yield

**DOI:** 10.1186/1754-6834-7-11

**Published:** 2014-01-22

**Authors:** Ajaya K Biswal, Kazuo Soeno, Madhavi Latha Gandla, Peter Immerzeel, Sivakumar Pattathil, Jessica Lucenius, Ritva Serimaa, Michael G Hahn, Thomas Moritz, Leif J Jönsson, Maria Israelsson-Nordström, Ewa J Mellerowicz

**Affiliations:** 1 Department of Forest Genetics and Plant Physiology, Swedish University of Agricultural Sciences, S901 83 Umeå, Sweden; 2 Department of Chemistry, Umeå University, S901 87 Umeå, Sweden; 3 Department of Physics, University of Helsinki, POB. 64FI-00014 Helsinki, Finland; 4 Complex Carbohydrate Research Center, BioEnergy Science Center (BESC), University of Georgia, 315 Riverbend Rd, Athens, GA30602-4712 USA; 5 Present address: National Agricultural Research Center for Western Region, National Agriculture and Food Research Organization (NARO), Zentsuji, Kagawa 765-8508 Japan

**Keywords:** *Populus*, Wood development, Secondary cell wall, Lignocellulose, Biofuel, Pectin

## Abstract

**Background:**

Wood cell walls are rich in cellulose, hemicellulose and lignin. Hence, they are important sources of renewable biomass for producing energy and green chemicals. However, extracting desired constituents from wood efficiently poses significant challenges because these polymers are highly cross-linked in cell walls and are not easily accessible to enzymes and chemicals.

**Results:**

We show that aspen pectate lyase PL1-27, which degrades homogalacturonan and is expressed at the onset of secondary wall formation, can increase the solubility of wood matrix polysaccharides. Overexpression of this enzyme in aspen increased solubility of not only pectins but also xylans and other hemicelluloses, indicating that homogalacturonan limits the solubility of major wood cell wall components. Enzymatic saccharification of wood obtained from PL1-27-overexpressing trees gave higher yields of pentoses and hexoses than similar treatment of wood from wild-type trees, even after acid pretreatment.

**Conclusions:**

Thus, the modification of pectins may constitute an important biotechnological target for improved wood processing despite their low abundance in woody biomass.

## Background

There is high interest in cultivating *Populus* species as energy crops because they grow rapidly, producing abundant lignocellulosic biomass that can be used as feedstock for biofuel and biomaterial production [1]. However, a major challenge hindering large-scale commercial use of *Populus* lignocellulose is its recalcitrance to degradation by bacterial and fungal enzymes, which complicates the separation of non-crystalline carbohydrate polymers and cellulose contents from lignin, and their subsequent saccharification. The constitutive lignocellulose polymers are non-randomly arranged within cell-wall layers that each have a distinct composition and architecture [2]. The outermost thin layer of middle lamella and primary cell wall, frequently referred to as the compound middle lamella, is rich in pectin and xyloglucan (XG) and is heavily lignified, whereas the inner thicker secondary-wall layers are enriched in cellulose and in hemicelluloses such as xylans and mannans, but are thought to contain relatively less lignin. Secondary-wall layers comprise the bulk of wood biomass and are traditionally believed to govern wood properties. However, hydrolysis of XG, a primary wall-layer hemicellulose in *Populus*, has been unexpectedly shown to affect solid wood traits [3] and accelerate the lignocellulose saccharification [4]. Moreover, a recent study has shown that alleles of the *Eucalyptus* genes *PME6* and *PME7*, encoding pectin methyl esterases, are associated with solid-wood quality properties, suggesting that pectin structure significantly influences wood properties [5].

Pectins in wood cell walls are composed mainly of homogalacturonan (HG) and rhamnogalacturonan I (RGI), which are concentrated in a thin outermost coat of the compound middle lamella, and have not been reported in xylan-containing secondary-wall layers [2]. The backbones of different pectin polymers are thought to be covalently connected, forming a supramolecular network linked to other cell-wall polymers via unknown bonds [6]. A recent study has identified a low abundance proteoglycan covalently linking pectins, arabinogalactan (AG) and arabinoxylan [7]. The final structure of the pectin network with its associated polymers is determined by activities of wall-residing trans-glycosylases, esterases, hydrolases and pectate lyases (PELs) [8].

Plant PELs (EC 4.2.2.2), which belong to polysaccharide lyase family 1 (PL1) (http://www.cazy.org), cleave the α-1,4 glycosidic bond between the galacturonic acid units of HG by β-elimination and release unsaturated oligogalacturonides. Genome-wide expression analyses in *Arabidopsis* have suggested that this gene family is involved in growth, cell adhesion and primary cell wall decomposition [9,10]. A PEL, encoded by *ZePel*, also appears to be involved in the differentiation of tracheary elements in *Zinnia elegans*[11]. In *Populus*, PEL genes have been assigned the CAZy family name PL1 and consecutive numbers *PL1-1* to *PL1-28*[12]. Many of these genes are highly expressed during xylem cell expansion [13]. *PtxtPL1-27* has been found to be highly upregulated in transgenic hybrid aspen (*Populus tremula* L. *x tremuloides* Michx.; *Ptxt*) with enhanced secondary growth caused by overexpression of GA20 oxidase [14]. Here we present a biochemical characterization of the recombinant *Ptxt*PL1-27 protein and an analysis of the effects of its overexpression in hybrid aspen. The results indicate that *Ptxt*PL1-27 can substantially modify the extractability of polymers from xylem cell walls, enhancing saccharification. Although pectin degradation has been shown previously to enhance saccharification of stems in herbaceous plants [15], it was surprising to find that it also affects saccharification in the case of aspen wood. Our analyses showed however that these effects in aspen are qualitatively and quantitatively different than those reported for herbaceous plants.

## Results and discussion

**Figure 1:**
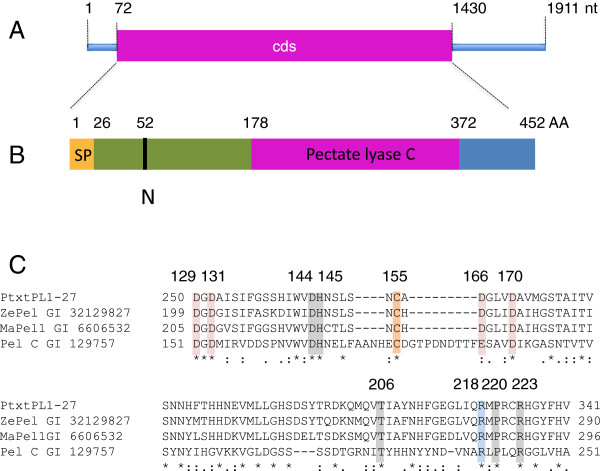
**Structural features of *Ptxt*PL1-27. (A)** Structure of mRNA [GenBank: EU379971.1]. Cds, coding sequence; nt, nucleotides. **(B)** Structure of the deduced peptide showing the signal peptide (SP), the pectate lyase C catalytic domain, and the predicted N-glycosylation site (N). AA, amino acids. **(C)** A multiple alignment of the catalytic site of *Ptxt*PL1-27 and pectate lyases of known enzymatic activities, showing the conserved residues involved in Ca^+2^ binding (pink), disulfide bonds (orange), catalysis (blue), and substrate binding (gray) with numbering according to Pel C structure as in [16]. ZePel, *Zinnia elegans* pectate lyase; MaPel1, *Musa acuminata* pectate lyase 1; Pel C, *Erwinia chrysanthemi* pectate lyase C.

An EST clone, A053p77u [GenBank: AI164054], of hybrid aspen corresponding to the *Populus trichocarpa* gene *PtPL1-27*[12] represented the most upregulated gene in a microarray study of fast-growing GA20 oxidase overexpressing lines [14]. The corresponding 1911 nucleotide (nt)-long transcript was isolated by 5′ RACE [GenBank: EU379971.1.] and its length was confirmed by northern blot analysis (Additional file 1). The isolated transcript contains an open reading frame of 1359 nt and has 99.6% identity at the amino acid level to Pt*PL1-27* (Figure 1A). Thus, we named this cDNA *Ptxt*PL1-27. The deduced mature protein encoded by the cloned cDNA has a molecular mass of 47 kDa, an isoelectric point of 7.75 (Figure 1B,C), and contains all of the conserved residues of PELs known to be involved in Ca^2+^-binding, substrate binding and catalysis [16].

**Figure 2:**
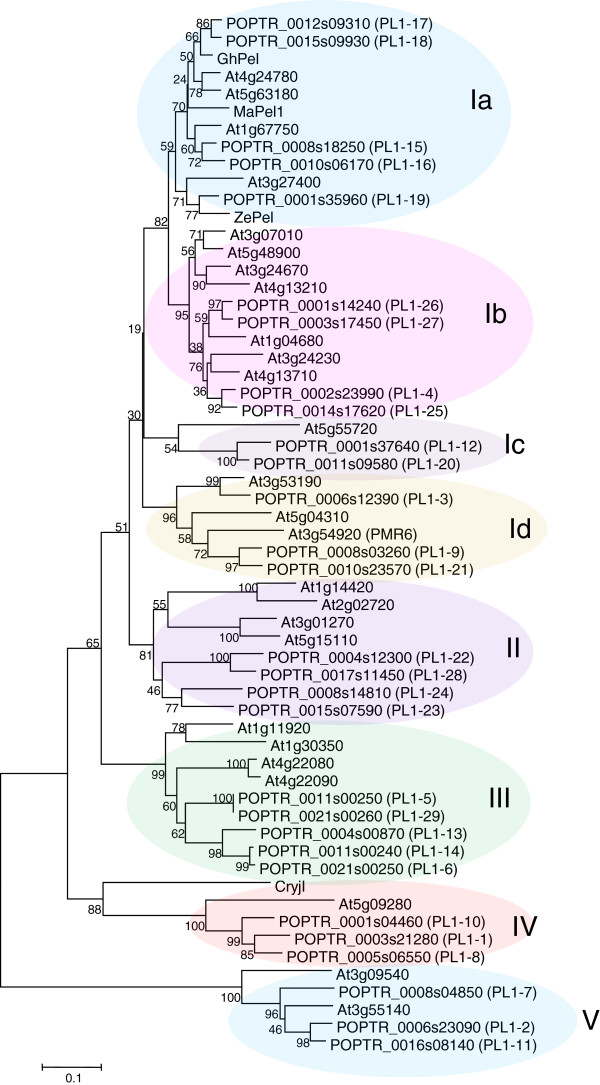
**Phylogenetic tree of pectate lyases in *Arabidopsis thaliana*, *Populus trichocarpa*, and other plants.** The tree was constructed by the neighbor-joining method using MEGA5*.* The *Populus* gene numbering is consistent with [12] and family designation (I-V) is consistent with [10]. The optimal tree with the sum of branch length = 5.9 is shown. The percentage of replicate trees in which the associated taxa clustered together in the bootstrap test (1,000 replicates) are shown next to the branches. Cryj1, *Cryptomeria japonica* pectate lyase [GenBank: BAA05542]; GhPel, *Gossypium hirsutum* pectate lyase [GenBank: ADB90478]; MaPel1, *Musa acuminata* pectate lyase 1 [GenBank: AAF19195]; ZePel, *Zinnia elegans* pectate lyase [GenBank: Y09541].

**Figure 3:**
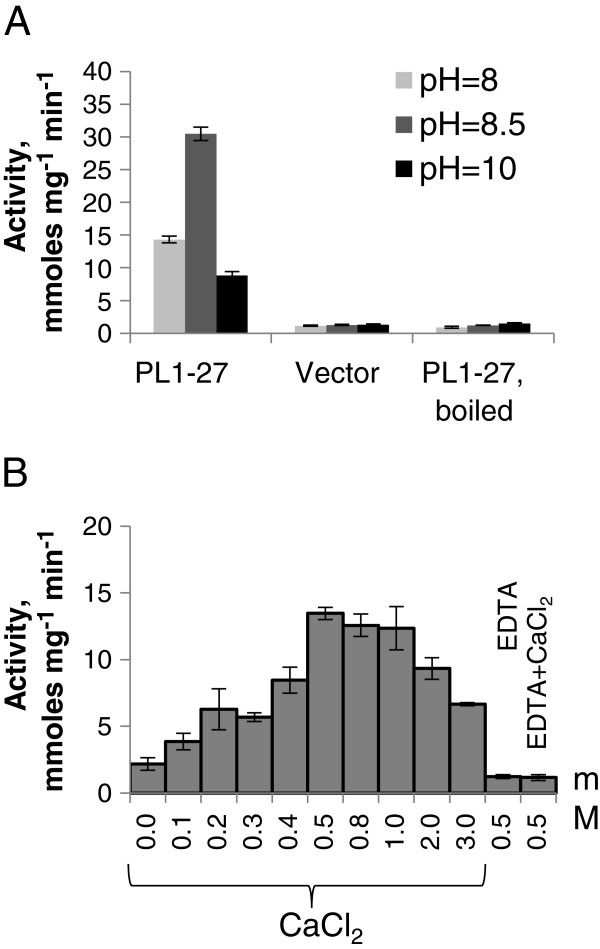
**PEL activity of *Ptxt*PL1-27 expressed in *Escherichia coli*.** PEL activity was measured as the formation of unsaturated uronides. Effect of pH **(A)** and Ca^2+^ ions **(B)**. Means of five replicates ± standard error. Vector denotes the proteins extracted from the bacteria expressing the vector without *Ptxt*PL1-27. EDTA, ethylenediaminetetraacetic acid.

Phylogenetic analysis of 26 *Arabidopsis thaliana*[10] and 29 *Populus trichocarpa* PL1 members (Additional file 2) revealed five major clades, with clade I further divided into four sub-clades (Figure 2). PL1-27 belongs to clade Ib. Plant PEL-like genes encoding proteins with experimentally validated PEL activity are members of clades Ia (ZelPel [11]), *Ma*Pel1 [17], *Gh*Pel [18]) or clade IV (Cry j I [19]). To characterize the enzymatic activity of *Ptxt*PL1-27, we expressed the mature protein in *Escherichia coli* and partially purified it by affinity chromatography (Additional file 3). The purified protein displayed PEL activity (4.2.2.2) towards polygalacturonic acid substrate, releasing 30 mmol of unsaturated uronides min^-1^ mg^-1^ at pH 8.5 (Figure 3). This is comparable to the specific activity of cotton *Gh*Pel [18], and much higher than that of *Zel*Pel [11], CryJ I [19] and *Ma*Pel1 [17]. As is typical for PELs of family PL1 [20], the PEL activity of *Ptxt*PL1-27 is optimal at basic pH, stimulated by Ca^+2^ and inhibited by ethylenediaminetetraacetic acid (EDTA).

**Figure 4:**
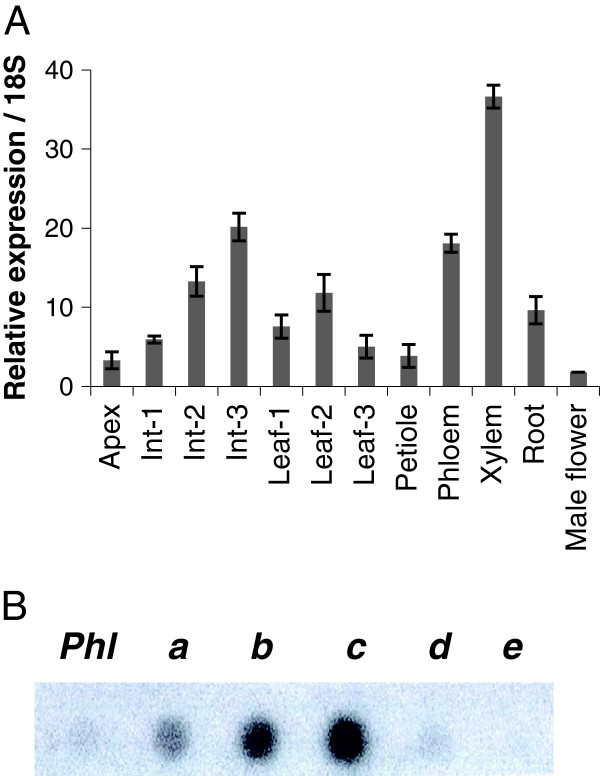
***Ptxt*PL1-27 expression analysis in aspen. (A)** Real time RT-PCR analysis of *PtxtPL1-27* expression in different tissues and organs of hybrid aspen indicates the highest expression of this gene in developing xylem and organs containing this tissue. Data shown are means of two biological replicates ± standard error. **(B)** Expression of *PtxtPL1-27* in different wood development zones isolated by tangential sectioning. The same amount of total RNA from each zone was blotted onto a membrane and hybridized with a radiolabeled probe corresponding to *PtxtPL1-27* cDNA. Phl, phloem; **a**, vascular cambium; **b**, expansion zone; **c**, transition zone; **d**, secondary wall formation zone; **e**, cell death zone.

Real time RT-PCR analysis was used to determine *PtxtPL1-27* transcript distribution in different organs and tissues of hybrid aspen. The analysis showed the highest expression in developing secondary xylem (Figure 4A). In the stem, the expression was higher in fully elongated internodes than in partially elongated internodes. Transcript profiling of the cambial region by tangential sectioning revealed that expression was highest in the transition zone, where xylem cell expansion declines and secondary cell-wall deposition begins (Figure 4B). These data indicate that *PtxtPL1-27* is expressed at a later stage of xylem cell development than other known pectin-degrading enzymes [13], outside the region of the IAA peak [21] where major cell expansion takes place and cell walls are expected to have low pH as a result of IAA action [22]. This expression pattern, along with the high pH optimum for *Ptxt*PL1-27 enzymatic activity (Figure 3A), suggests that the enzyme may be involved in cell-wall modification during the late stage of xylem differentiation and even the secondary walled stage rather than in cell expansion.

**Figure 5:**
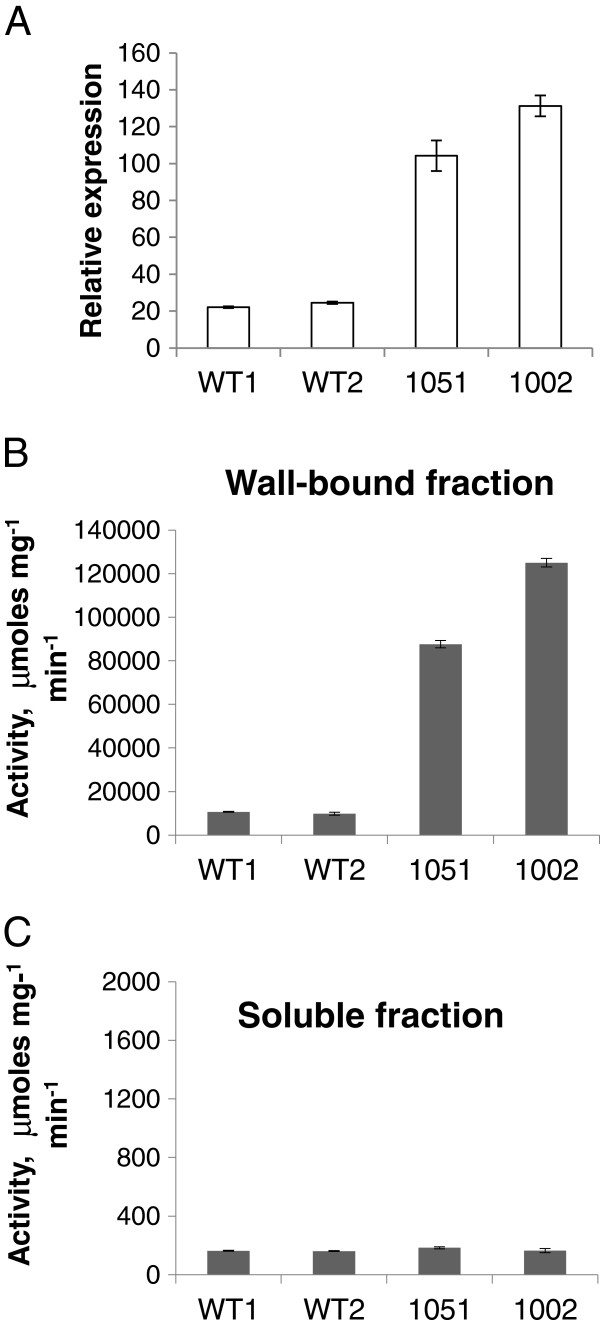
**Overexpression of *Ptxt*PL1-27 in developing wood of aspen.** Relative *PtxtPL1-27* transcript level determined by RT-qPCR and calibrated to 18S RNA **(A)** and PEL activity in wall-bound and soluble protein fractions **(B, C)** in developing xylem in the most highly expressing independent transgenic lines carrying 35S: *Ptxt*PL1-27 (1051, 1002) and wild-type (WT). Means of two experiments, each with three biological replicates. WT1 and WT2 represent two sets of randomly selected WT trees. Bars represent standard error.

**Figure 6:**
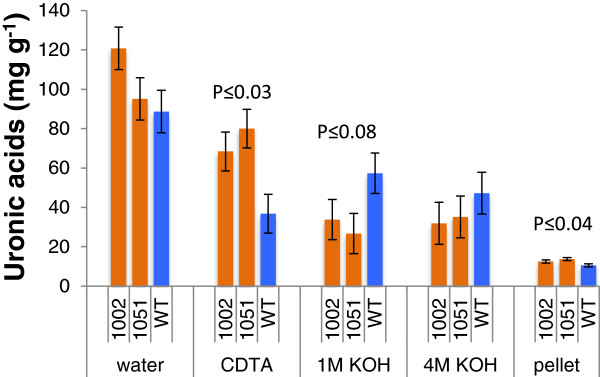
**Overexpression of *PtxtPL1-27 *affects extractability of acidic polymers from wood.** Wood alcohol-insoluble residues (AIRs) were sequentially extracted with water, 1,2-cyclohexylenedinitrilotetraacetic acid (CDTA) 1 M potassium hydroxide (KOH) and 4 M KOH, and the amounts of uronic acids extracted by each solvent and remaining in the pellet was determined by the biphenyl assay and expressed per weight of extracted cell wall material. Data are means of three biological replicates and standard error. Probability refers to the post analysis-of-variance analysis by contrast between transgenic lines and wild-type (WT).

To investigate effects of *PtxtPL1-27* on developing wood, its coding sequence was overexpressed in aspen under the control of the CaMV 35S. The two lines that showed the highest expression levels out of the 20 lines that were obtained were selected for chemical wood analyses. Compared with wild-type (WT) counterparts, *PtxtPL1-27* transcript levels were five to six times higher in the secondary-walled developing xylem (Figure 5A), and PEL activity was ten to twelve times higher in the wall-bound protein fraction in samples from these lines, whereas no change in the soluble fraction was observed (Figure 5B,C). In addition, the ectopic expression of *PtxtPL1-27* resulted in growth inhibition (Additional file 4) similar to that observed after ectopic overexpression of polygalacturonase [15]. To account for differences in growth rates between the lines, wood samples were taken from the same internodes (40 to 44) to compare samples of the same cambial age in plastochrons. Basic wood chemical analyses did not reveal any major changes in the transgenic lines, only minor increases in lignin and trifluoroacetic acid (TFA)-soluble Glc contents, and a small decrease in Man contents (Table 1). Contrary to our expectations based on the activity of *Ptxt*PL1-27, we did not observe significant reduction in the uronic acid (UA) contents of the wood cell walls in the transgenic lines (Table 1). It is possible that there is a substantial fraction of glucuronic acid from glucuronoxylan in total wood uronic acids [23], and therefore changes in galacturonic acid in the pectin fraction of the transgenic lines are proportionally small and remain undetected. On the other hand, it is also possible that HG, after being cleaved by PEL, remains in cell walls and is held by interacting polymers. In apple leaves, the overexpression of PG did not lead to overall decrease in UAs but to increased HG extractability [24]. Therefore, we examined UA contents in pectin fractions sequentially extracted from cell walls by water and 1,2-cyclohexylenedinitrilotetraacetic acid (CDTA) 1 M potassium hydroxide (KOH) and 4 M KOH, and their contents remaining in the residual wall pellets in the transgenic lines and in WT (Figure 6). Significantly higher content of UAs in the CDTA extract and a lower content in the 1 M KOH extract were recorded in the transgenic lines compared to WT. These results suggest that the overexpressed PEL induced important changes in extractability of uronic acid-containing polymers.

**Table 1:** Lignin, hydrolyzable sugar anhydrate content and crystalline cellulose content of aspen wood

	**Wild-type**	**1051**	**1002**
Klason lignin	**16.8 ± 0.2**^1^*	17.7 ± 0.3**	17.1 ± 0.3
Acid soluble lignin	2.1 ± 0.0	2.1 ± 0.0	2.0 ± 0.1
Cellulose	364 ± 28	346 ± 62	363 ± 28
Rha	11.7 ± 1.2	9.2 ± 0.6	9.0 ± 1.2
Ara	5.6 ± 0.1	6.8 ± 0.4	6.1 ± 0.5
Xyl	312.4 ± 5.7	304.1 ± 6.4	317.8 ± 2.1
Man	20.5 ± 0.4**	18.8 ± 0.6	16.1 ± 0.9**
Gal	11.5 ± 0.9	13.3 ± 0.5	13.1 ± 1.0
Glc	178.8 ± 0.4*	187.2 ± 2.3	191.9 ± 4.8
Total sugar	540.4 ± 7.4	539.4 ± 5.3	554.1 ± 6.8
Uronic acids	31.8 ± 2.4	30.4 ± 2.0	27.9 ± 1.5

Lignin content is expressed in % (w/w). Sugar content is expressed in μg/mg of wood de-starched alcohol-insoluble residue (AIR). Means ± standard error, n = 10 or 2 biological replicates for lignin and sugars, respectively.

^1^Averages for the transgenic lines in bold are significantly different from wild-type (WT) (post analysis-of-variance (ANOVA) *t*-test). Averages for WT in bold are significantly different from both transgenic lines taken together (post-ANOVA contrast). *P ≤10%; **P ≤5%.

**Figure 7:**
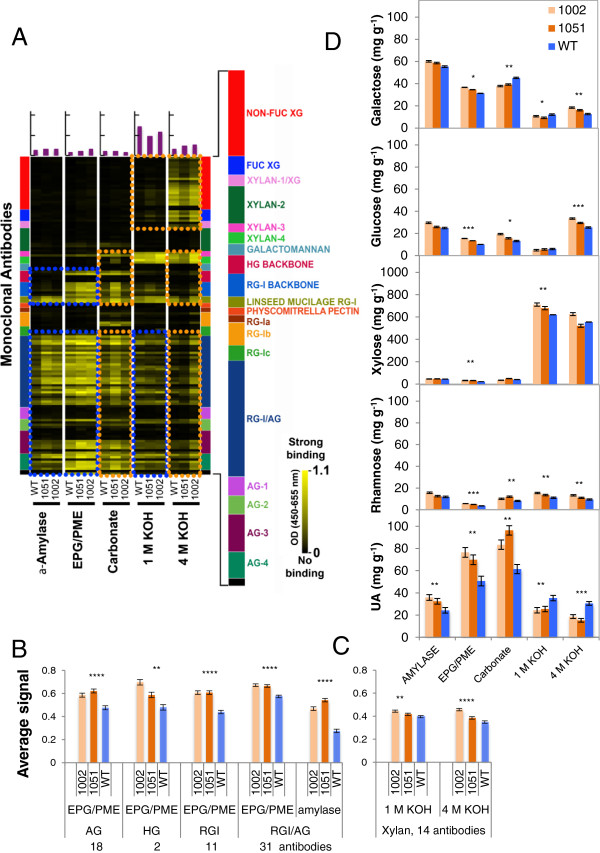
**Overexpression of *PtxtPL1-27 *affects extractability of many polymers of wood.** Sequential cell wall extracts, obtained using α-amylase, endo-polygalacturonase (EPG)/ pectin methylesterase (PME), sodium carbonate, 1 M potassium hydroxide (KOH) and 4 M KOH, were probed with a panel of 150 monoclonal antibodies **(A-C)** and analyzed for the monosaccharide composition **(D)**. **(A-C)** Extracts were screened against an array of plant glycan-directed monoclonal antibodies using ELISA. **(A)** Antibody binding (average of two biological replicates) is depicted as colored heat maps; bright yellow, maximal binding; black, no binding. The colored panel (right) depicts groups of antibodies used, identified according to polysaccharides predominantly recognized by each group [26] (list of antibodies provided in Additional file 5). Colored boxes indicate regions of the glycome profiles that were altered in both transgenic lines (blue boxes) or predominantly in one line (orange boxes) compared to wild-type (WT). **(B)** Signals ≥0.1 from pectin- and arabinogalactan (AG)-directed antibodies recognizing epitopes in α-amylase and EPG/PME extracts. **(C)** Signals ≥0.1 from an expanded set of xylan-directed antibodies specifically recognizing epitopes in aspen wood hemicelluloses in 1 M KOH and 4 M KOH extracts. **(D)** Monosaccharide composition of sequential extracts. Only monosaccharides that differed in content in extracts between *PtxtPL1-27* overexpressing lines and the WT are presented (full dataset in Additional file 6). Data in **B-D** are means of two biological replicates ± standard error. Asterisks indicate probability for significance of difference between transgenic lines and WT (post analysis-of-variance contrast: *P ≤10%, **P ≤5%, ***P ≤1%, ****P ≤0.1%).

To identify the polymers with extractability that was affected by PEL overexpression, we applied antibody-based glycome profiling [25-28] to the sequential extracts of wood from *Ptxt*PL1-27-overexpressing and WT saplings, prepared using α-amylase, endo-polygalacturonase (EPG)/pectin methylesterase (PME), CaCO_3_, 1 M KOH and 4 M KOH. Signals from most of 150 monoclonal antibodies used in the profiling ([26]; Additional file 5) showed clear qualitative differences between genotypes (Figure 7A). The main differences between the two overexpressing lines and WT plants were the increased signals for many typical epitopes of pectins and AGs in the α-amylase and/or EPG/PME extracts (and in some cases, weaker or stronger in 1 M KOH fractions) of the transgenics (blue boxes; Figure 7A), strongly indicating that overexpression of *Ptxt*PL1-27 increased the extractability of these polymers. The signals stronger than optical density (OD) = 0.1 from these polymers were statistically analyzed, and significant changes are shown in Figure 7B. The increased amounts of HG, RGI and AG epitopes in EPG/PME extracts are supported by the increased contents of UAs, Rha and Gal in this fraction (Figure 7D). Other differences were noted in comparing the glycome profiles of the overexpressing lines with WT, particularly in the carbonate and 4 M KOH extracts (orange boxes, Figure 7A). In particular, higher signals with xylan-directed antibodies in line 1002 were noted in the 4 M KOH extract. However, these differences were not always consistent between the two overexpressing lines, making it difficult to correlate these changes directly with the overexpression of *Ptxt*PL1-27. It was also evident that few of the anti-xylan antibodies included in these initial studies reacted strongly with aspen hemicelluloses. To address the question of xylan extractability, we expanded the panel of xylan-directed antibodies (http://www.wallmabdb.net), and analyzed aspen 1 M KOH and 4 M KOH extracts. Fourteen of these xylan-directed antibodies bound strongly to WT aspen hemicellulose extracts (OD ≥0.1) (Additional file 5). Average ELISA signals of these monoclonal antibodies showed increases in transgenic lines in both 1 M KOH and 4 M KOH extracts compared to the corresponding extracts from WT walls (Figure 7C), indicating increased extractability of xylan. These results are in agreement with the changes seen in xylose content in the 1 M KOH extract (Figure 7D and Additional file 6). In summary, the glycome profiling of sequential wall extracts indicated increased solubility of pectins, AGs and xylans in the transgenic lines.

In conclusion, the above cell-wall analyses revealed that PEL overexpression induced no major changes in the composition of cell walls in the wood, but substantially altered extractabilities of pectins, AGs and xylans in the wood. These results concerning the cells with secondary cell-walls are reminiscent of the previously published data on primary-walled tissues. In strawberry fruits, the downregulation of PEL expression was found to reduce the solubility of UAs and neutral sugars [29]. Thus, in both primary and secondary walls, the integrity of an enzyme-accessible HG domain appears to influence the mobilization of major cell-wall matrix components (HG, RGI, xylan and AG). Several previous studies have suggested that pectins and hemicelluloses are cross-linked in primary walls [28-34]. A covalent linkage between RGI, HG, and arabinoxylan involving an AG protein has been identified recently in the Arabidopsis cell suspension culture medium [7]. It is possible that similar covalent linkages bind HG to AG and glucuronoxylan in the wood cell walls of aspen, and therefore the fragmentation of HG by PEL increases solubility of xylan and AG (Figure 7). Another possibility is that PEL activity alters pectin structure in a way that influences lignin polymerization, either by affecting lignin nucleation sites or activities of laccases or peroxidases [35,36]. Slightly increased Klason lignin contents were seen in the transgenic lines (Table 1) and might have affected polysaccharide extractability. Yet another possibility is that PEL increases cell wall porosity, thus facilitating access of native cell-wall endohydrolases to their substrates [8], thereby making these polymers more soluble.

**Figure 8:**
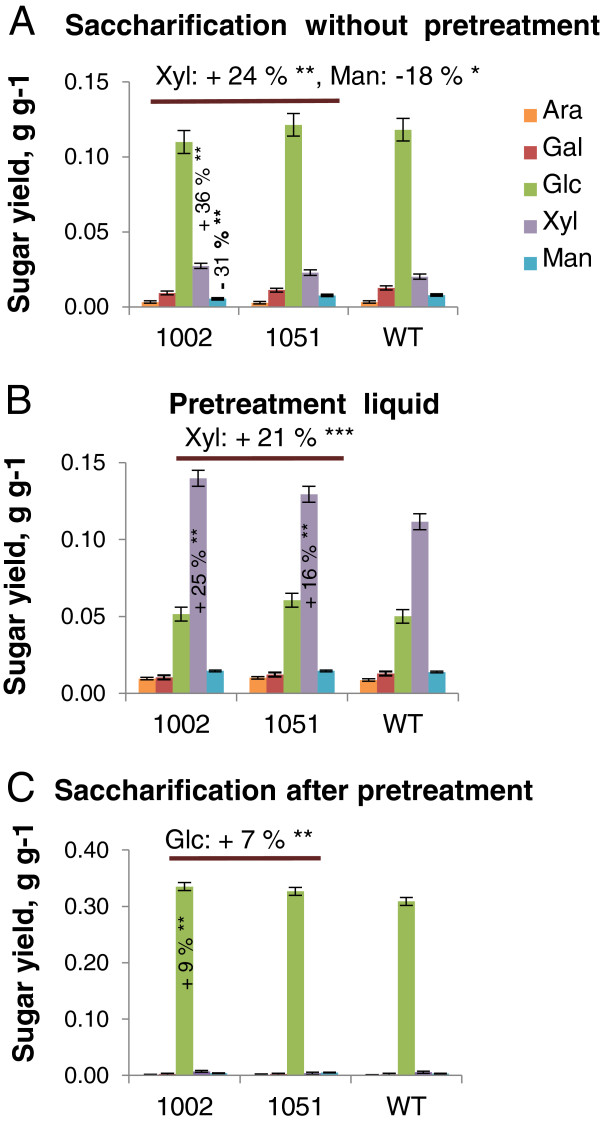
**Overexpression of *Ptxt*PL1-27 increases sugar yields from saccharification.** Sugar yields after 72 h of saccharification without pretreatment **(A)**, after acid pretreatment **(B)**, and after 72 h saccharification following acid pretreatment **(C)**. Means of five biological replicates ± standard error. Percentage differences are shown for individual lines significantly differing from wild-type (WT) (post analysis-of-variance (ANOVA) *t*-test), and average differences for both transgenic lines when significantly different from WT (post ANOVA contrast between transgenic lines and WT) *P ≤10%, **P ≤5%, ***P ≤1%.

To test the possibility that altered extractability of xylan and other matrix polymers improves the digestibility of lignocellulose, milled wood of the transgenic and WT lines was enzymatically hydrolyzed with and without acid pretreatment. Without pretreatment, glucose yields from the transgenic lines were not altered, but their xylose yields were 24% higher than WT yields (Figure 8). An 18% reduction in mannose yield was also observed, probably reflecting a similar reduction in mannose levels in the starting material (Table 1) rather than a change in mannan digestibility. Acid pretreatment resulted in the release of 21% more xylose, and subsequent enzymatic digestion yielded 7% more glucose from transgenic lines than from WT. As the liquid fraction obtained after pretreatment was removed before enzymatic hydrolysis of cellulose and as no increase in the cellulose content of the transgenic lines was detected (Table 1), the increased glucose yield after pretreatment (Figure 8C) could mainly be attributed to improved digestibility of cellulose. Overall, the combined yields of pentoses, hexoses and all monosaccharides from acid pretreatment and enzymatic saccharification were increased by up to 24 ± 0.08%, 8 ± 0.03% and 11 ± 0.02%, respectively (Table 2). These results contrast with those obtained with herbaceous plants that ectopically express fungal polygalacturonase or plant pectin methyl esterase inhibitor [15], in which saccharification efficiency was increased without pretreatment, but not when acid pretreatment was applied. In further contrast to our results, the increased saccharification (without pretreatment) in material from herbaceous plants was due to increases in glucose yields whereas xylose yields were not affected. It is likely that in primary walls, the removal of pectin increases accessibility of cellulose to enzymes. The accessibility of cellulose in secondary walls has been shown to be strongly influenced by their xylan and lignin contents [37-39], and xylan acid hydrolysis yields are reportedly inversely correlated to the syringyl/guaiacyl (S/G) ratio [40]. However, the effects of pectin structure and composition on these variables have not been studied previously in woody plants. This study shows the positive effect of pectin degradation on saccharification in woody tissues (Figure 8, Table 2).

**Table 2:** Total sugar yields for transgenic aspen lines and wild-type after acidic pretreatment

**Line**	**Total sugar yields (pretreatment liquid and enzymatic hydrolysate)**		
	**Y**_**Hexoses/W **_**(g g**^**-1**^**)**^**a**^	**Y**_**Pentoses/W **_**(g g**^**-1**^**)**^**b**^	**Y**_**Total/Wv**_**(g g**^**-1**^**)**^**c**^
**Wild-type**	0.390 ± 0.01 (100%)**	0.126 ± 0.01 (100%)***	0.517 ± 0.01 (100%)***
**1051**	0.421 ± 0.01 (108%)**	0.144 ± 0.01 (114%)**	0.565 ± 0.01 (109%)**
**1002**	0.418 ± 0.03 (107%)	0.157 ± 0.01 (124%)**	0.574 ± 0.01 (111%)**

The % values represent the sugar yield in comparison with wild-type (WT). Means ± standard error, n = 5 biological replicates. ^a^Yield of hexoses (g of Gal, Glc, and Man) per g of wood after pretreatment and after 72 h of enzymatic hydrolysis. ^b^Yield of pentoses (g of Ara and Xyl) per g of wood after pretreatment and after 72 h of enzymatic hydrolysis. ^c^Yield of monosaccharides (g of Ara, Gal, Glc, Man and Xyl) per g of wood after pretreatment and after 72 h of enzymatic hydrolysis. Asterisks (by means for individual lines) indicate yields significantly different from WT when analyzed by post analysis-of-variance (ANOVA) *t*-test or means (for WT) that were significantly different from both lines by post ANOVA contrast. **P ≤5%, ***P ≤1%.

Since the transgenic lines gave higher enzymatic glucose yields and had slightly higher TFA-soluble glucose contents, we hypothesized that cellulose crystallinity may be affected by *PtxtPL1-27* overexpression. To test this hypothesis, crystallite structures in the wood of transgenic and WT lines were examined by x-ray diffraction. However, no major differences in either the crystallite width or overall crystallinity were observed (Table 3). Thus, the higher glucose yields could be partly explained by an increase in TFA-soluble glucan contents and partly by increased accessibility of cellulose due to better xylan extraction during pretreatment (Figure 8B). The latter effect might be directly related to increased solubility of xylan observed in the transgenic lines (Figure 7). Another likely possibility is that PEL activity erodes the outer pectin coat of middle lamella, thereby increasing access of chemicals and enzymes to secondary walls. Interestingly, the relative increase in glucose yields was smaller compared to that on xylose yields. Direct application of pectin-degrading enzymes (EPG/PME) to woody lignocellulose from poplar has been also shown to solubilize xylan, but the subsequent cellulose digestion was not affected by this treatment [41]. These results suggest that the effects of pectin degradation might differ between *in vitro* and *in vivo* conditions.

**Table 3:** Cellulose crystallite structure in transgenic lines and wild-type determined by x-ray diffraction

**Line**	**Crystallinity %**	**Crystallite width Å**
Wild-type	28.7 ± 1.5	29.9 ± 0.1
1051	29.4 ± 1.8	30.0 ± 0.2
1002	30.7 ± 1.7	30.2 ± 0.1

Means ± standard error, n = 5 to 8 biological replicates. Å – ångström.

## Conclusions

In overall conclusion, we have shown that *Ptxt*PL1-27 from clade Ib of the PL1 family is a PEL, which is expressed in differentiating xylem at the onset of secondary wall formation. When overexpressed, it is capable of loosening several matrix components from secondary-walled xylem cells without significantly affecting their overall cell wall composition. PEL activity is beneficial for lignocellulose saccharification, increasing yields of monosaccharides, especially xylose, with and without pretreatment. Thus, pectin structure may affect wood saccharification even though it is a minor wood component. These findings could be exploited in several ways. First, our results could be used to design more efficient saccharification cocktails for deconstruction of woody biomass. Second, our results suggest that targeting pectin in the genetic improvement programs for woody feedstocks might prove beneficial for subsequent processing and deconstruction of woody biomass. Third, genetic engineering targeted toward pectin-modification in woody plants might improve their suitability as feedstock for biofuel production. They also show that the genetic engineering strategy would need to be targeted to mature woody tissues in order to avoid negative effects on plant growth and development that have been observed in this and other studies [15,42].

## Materials and methods

### Cloning of *PtxtPL1-27*

Full-length cDNA was cloned from the hybrid aspen cDNA from elongating shoots by 5′ RACE using Marathon (Clontech Laboratories Inc., Mountain View, CA, USA), and the gene-specific primer Race1 (Tab. S3) based on AI164054. The complete cDNA was reconstructed by PCR using Advantage PCR Cloning Kit (Clontech) with F1 and R1 primers that included BamHI restriction sites (Additional file 7) and hybrid aspen cDNA. The PCR product was sub-cloned, sequenced and named *PtxtPL1-27* [GenBank: EU379971.1].

### Heterologous expression and enzyme assays

Mature *Ptxt*PL1-27 peptide (**Δ**_1-26_*Ptxt*PL1-27) was expressed in *E. coli* (ER2566) using the pTYB11 vector (IMPACT™-CN System, New England Biolabs Inc., Ipswich, MA, USA) carrying the PCR product obtained with the primers MF1 and MR1 (Additional file 7). Cells containing the recombinant plasmid were grown in LB medium with 100 μg/ml carbenicillin to an OD of A_600_ = 0.5. Expression of the recombinant gene was then induced by incubating the cells with 0.3 mM isopropyl β-D-thiogalactoside (IPTG) at 14°C for 15 h. The cells were resuspended in 20 mM 4-(2-hydroxyethyl)piperazine-1-ethanesulfonic acid (HEPES) 500 mM NaCl and 1 mM EDTA, pH 8.0, lysed by sonication on ice, and centrifuged at 20,000 g for 30 minutes at 4°C. The pellet was dissolved in the same buffer and purified using an IMPACT™-CN chitin beads column (New England Biolabs). PEL activity (EC 4.2.2.2) was assayed in the column-purified protein extract according to Collmer *et al.*[43] using 0.24% w/v of polygalacturonic acid (Sigma-P3889) in 60 mM Tris–HCl, pH 8.5 (unless otherwise indicated), 0.5 mM CaCl_2_, and 0.3 ml of protein extract in 1.5 ml reaction mixtures. The unsaturated uronide products were measured by absorbance at 235 nm over 180 minutes at 30°C using the molar extinction coefficient 4.6 × 10^3^ M^-1^ cm^-1^. Specific activity was expressed per mg of protein as determined by a Bio-Rad Assay kit (Life Science, Sunbyberg, Sweden) using BSA as a protein standard.

### Generation of transgenic aspen lines

The full-length *PtxtPL1-27* cDNA clone lacking 5′ and 3′ untranslated regions (UTRs) was released by digestion with XbaI and SalI and ligated in sense orientation into the plant binary vector pMH1.kana (a gift from Mattias Holmlund, SLU, Umeå, Sweden) under control of the CaMV 35S promoter. The construct was transformed into hybrid aspen clone T89 by *Agrobacterium*-mediated transformation, and plants were regenerated, planted in the greenhouse and grown for approximately three months, as previously described [42]. Briefly, the growing conditions were: 18-h photoperiod, a temperature regime of 22°C/17°C (day/night), and a relative humidity of 70%.

### Analysis of RNA in the transgenic and WT lines

RNA was isolated from developing xylem scraped from the wood surface after peeling the bark, cambium and phloem scraped from the exposed bark surface, root tips approximately 0.5 cm long, internodes up to 50%, 50 to 80%, and 100% of their final length, designated internodes 1, 2 and 3, leaves subtending these internodes (leaf 3 was fully expanded), the apical bud and petioles from internodes 1 and 2, pooled. In addition, we extracted RNA from a series of samples of tangentially sectioned developing wood. Total RNA was extracted by hexadecyl-trimethylammonium bromide (CTAB) and purified as previously described [42].

cDNA was synthesized from the total RNA samples using a blend of oligo (dT) and random primers and an iScript™ cDNA Synthesis Kit (Bio-Rad, Hercules, CA, USA). Dot blot was prepared as described previously [42] and probed under stringent conditions with a probe based on AI164054. Primers for real-time PCR were designed using Beacon Designer (v 2.1, Premier Biosoft International, Palo Alto, CA, USA) and tested for efficiency and specificity. The selected primers are listed in Additional file 7. PCR reactions were performed in triplicate using a Bio-Rad iCycler MyiQ Real-Time PCR Detection System and products were detected with SYBR Green (iQ SYBR Green Supermix). The threshold cycle (Ct) was calculated using iCycler MyiQ software 1.0 (Bio-Rad, Hercules, CA, USA). The PCR efficiencies (E) were determined using 4-fold serial dilutions of pooled cDNA originating from the assayed tissues. Relative transcript levels were then calculated as:

$$100,000\times {E^{C}}^{t\;\mathrm{Control}}/{E^{C}}^{t\;\mathrm{Target}}$$

### Protein extraction and PEL activity in aspen

Proteins were extracted from scraped xylem from internodes 44 to 60: 5 g of tissue were ground in liquid N_2_ to a fine powder, stirred for 30 minutes at 4°C in 25 ml of buffer A (50 mM sodium phosphate, pH 7.0, 2 mM EDTA, 4% polyvinylpyrolidone (PVP) mw 360 000, 1 mM dithiothreitol (DTT), and centrifuged at 10,000 rpm for 10 minutes at 4°C. The supernatant was collected as the soluble fraction and the pellet was re-suspended in 25 ml of buffer A supplemented with 1 M NaCl, stirred for 30 minutes at 4°C, centrifuged as above, and the supernatant was collected as the wall-bound fraction. Saturated ammonium sulfate (AS) was added to reach 20% and 19% of saturation, to the soluble- and wall-bound fractions, respectively, stirred for 30 minutes at 4°C, and centrifuged as above to remove PVP from solution. Solid AS was added to the supernatants, increasing the AS saturation to 90% and 80% for the soluble- and wall-bound fractions, respectively, and stirred for 30 minutes at 4°C to precipitate proteins. Following the centrifugation as above, the pellets were dissolved in 1 ml TE buffer, pH 8.0. The dissolved proteins were desalted with PD10 columns and protein concentrations were determined by the Bradford method using the Bio-Rad Assay kit (Life Science). Pectate lyase activity was assayed as described above using 0.3 ml of extracted proteins.

### Wood chemical analyses

Wood of internodes 40 to 44 was freeze-dried and ground using an A11 Basic Analytical Mill (IKA, Staufen, Germany) and then using an Ultra Centrifugal Mill ZM 200 equipped with a 0.5 mm ring sieve (Retsch) at 30 Hz for 150 sec. The contents of acid-resistant lignin (Klason lignin) and acid-soluble lignin were determined according to Theander and Westerlund [44].

Alcohol-insoluble residue (AIR) was obtained by washing the ground wood sequentially in 70% ethanol, methanol:chloroform 1:1 (v/v) and acetone, then drying overnight under vacuum. Starch was removed by treatment with *Bacillus* α-amylase (Sigma - A6380) at 5,000 units per g of AIR, 16 h at 37°C. Crystalline cellulose was purified by hydrolysing non-cellulosic polysaccharides with acetic acid:nitric acid:water (8:1:2, v/v/v) and removing the supernatant. The resulting pellet was washed four times with acetone, dried, hydrolysed in 72% sulphuric acid, and the glucose content was determined with the anthrone method [45].

Sequential extraction of the wood cell walls followed a previously published procedure [25]. The neutral glycosyl residue composition of non-fractionated and fractionated cell wall polysaccharides was analyzed by the alditol acetate method [46]. Briefly, after preparing alditol acetates, 1 μL samples were injected, splitless, into a Hewlett-Packard chromatograph 5890 (Ramsey, MI, USA) equipped with an SP 2330 (30 m × 0.25 mm, 0.25 μm film thickness, 24019 Supelco) column using helium as carrier gas, and the analytes were detected by a coupled mass spectrometer. UA contents of the cell wall polysaccharides were determined according to Filisetti-Cozzi and Carpita [47].

Cell wall extracts were diluted to 60 μg mL - 1 and 50 μL samples were used for glycome profiling, as previously described [25]. α-Amylase (type II-A from *Bacillus*) was obtained from Sigma (A6380), and EPG (I and II from *Aspergillus niger*) [48] and PME (from *A. niger*) [49] were donated by Carl Bergmann (Complex Carbohydrate Research Center, University of Georgia), and used at approximately 1.0 units/100 mg AIR in 50 mM sodium acetate, pH 5.0. Plant cell wall glycan-directed monoclonal antibodies [26] were obtained as hybridoma cell-culture supernatants either from laboratory stocks maintained by the Complex Carbohydrate Research Center (CCRC, JIM and MAC series; available from CarboSource Services (http://www.carbosource.net)) or from Fabienne Guillon (AX1 [27] (INRA, Nantes)).

### Saccharification

Freeze-dried wood from internodes 21 to 39 was milled as above, followed by sieving with an Analytical Sieve Shaker AS 200 (Retsch) to obtain 0.1- to 0.5-mm particles. Pre-pretreatment (if applied) consisted of incubation of 50 mg milled and sieved wood in 1% (w/w) sulfuric acid at 165˚C for 10 minutes under stirring in a single-mode microwave system (Initiator Exp, Biotage, Uppsala, Sweden). After acid pretreatment, the suspension was centrifuged, the liquid phase (the pretreatment liquid) was removed, and the pellet was washed. The remaining solid (pretreated wood), and the untreated wood powder (50 mg), were treated with 50 mg of a liquid enzyme mixture consisting of equal proportions of Celluclast 1.5 L (a liquid enzyme preparation from *Trichoderma reesei* and the main source of cellulase) and Novozyme 188 (a liquid enzyme preparation from *A. niger* and the main source of β-glucosidase) (both from Sigma-Aldrich, St. Louis, MO, USA) in sodium citrate buffer (50 mM, pH 5.2), using 1,000 mg as the total size of the reaction mixture, at 45°C for 72 h in an Ecotron orbital shaker (Infors, Bottmingen, Switzerland) at 170 rpm.

Concentrations of monosaccharides (Ara, Gal, Glc, Man, and Xyl) in the samples were then determined using an HPAEC system (ICS3000, Dionex, Sunnyvale, CA, USA) equipped with a Pulsed Amperometric Detection system and a CarboPac PA20 column (3 × 150 mm) with a CarboPac PA20 guard column (3 × 30 mm) (Dionex).

## Abbreviations

AG: arabinogalactan; AIR: alcohol-insoluble residue; ANOVA: analysis of variance; CDTA: 1,2-cyclohexylenedinitrilotetraacetic acid; EDTA: ethylenediaminetetraacetic acid; ELISA: enzyme-linked immunosorbent assay; EPG: endo-polygalacturonase; HG: homogalacturonan; KOH: potassium hydroxide; nt: nucleotide; OD: optical density; PEL: pectate lyase; PME: pectin methylesterase; RGI: rhamnogalacturonan I; SP: single nucleotide; TFA: trifluoroacetic acid; UA: uronic acid; WT: wild-type; XG: xyloglucan.

## Competing interests

The authors declare that they have no competing interests.

## Authors’ contributions

AKB participated in plant phenotyping, expression studies and cell wall analyses, carried out enzymology analyses, sequence alignment and helped to draft the manuscript. KS participated in generation of transgenic plants, phenotyping, and expression analyses. MLG participated in saccharification analyses. PI participated in cell wall analyses. SP participated in glycome profiling. JL participated in x-ray diffraction analysis. RS participated in x-ray diffraction analyses and in the design of research. MGH participated in glycome profiling and in the design of research. TM participated in the generation of transgenic lines and in the design of research. LJJ participated in saccharification analyses and in the design of research. MIN participated in generation of transgenic lines, phenotyping, expression analyses and in the design of research. EJM conceived the study, carried out statistical analyses, participated in the design, coordinated research and helped to draft the manuscript. All authors read, edited and approved the final manuscript.

## Supplementary Material

Additional file 1 **Detection of *PtxtPL1-27*** **transcript (EU379971.1) by northern blot.** Northern blot analysis showing the size of *PtxtPL1-27* transcript in the wild-type (WT) at 1.9 kb as compared to transcript size in four independent transgenic lines overexpressing *PtxtPL1-27* ORF (without untranslated regions (UTRs)) under control of the CaMV 35S promoter at 1.4 kb. A polyubiquitin probe was used as a reference control. The RNA used in the northern blot analysis originated from developing xylem tissue. Click here for file

Additional file 2 ***Populus trichocarpa *** **PL1 family genes.** The complete list of *Populus* PL1 family gene models based on *Populus trichocarpa* assembly version 2.2, including current names, former models, closest Arabidopsis homologues, corresponding *Populus* ESTs from Populus DB, and protein domains. Click here for file

Additional file 3 **SDS-PAGE showing the degree of purification of** Δ_**1-26**_ ***Ptxt*** **PL1-27 expressed in *E. coli. *** *Ptxt*PL1-27 protein was expressed in *E. coli* using pTYB11 vector and partially purified by affinity chromatography. Proteins were separated by gel electrophoresis and stained by Coomassie. **Lane 1:** proteins extracted from *E. coli* expressing recombinant protein before induction. **Lane 2:** proteins extracted from *E. coli* expressing recombinant protein after induction by 0.3 mM isopropyl β-D-thiogalactoside (IPTG). The induced product can be seen at 98.8 kDa polypeptide representing *Ptx*tPL1-27 fused to intein-chitin binding domain. **Lane 3:** partially purified recombinant protein is shown as 41.2 kDa band. The preparation is contaminated mainly by the 56.0 kDa polypeptide containing intein and chitin binding domain, which is the cleavage product of 98.8 kDa recombinant polypeptide. Click here for file

Additional file 4 **Ectopic overexpression of *PtxtPL1-27 *** **inhibits plant growth.** Data are relative stem increments and *PtxtPL1-27* expression levels in elongating stem internodes of independent transgenic lines. Stem-length increments were determined as an increase in stem length during 64 days of growth in the greenhouse. Between two and eight plants per line were measured. Expression was determined by RT-q-PCR using *18S* as a reference gene. Click here for file

Additional file 5 **Antibodies used for the ELISA-based glycome profiling.** The list of all monoclonal antibodies used in Figure 7. Click here for file

Additional file 6 **Monosaccharide composition of wood sequential extracts and the resulting pellets.** Sugar composition of wood extracts; means ± standard error, n = 2 biological replicates. Asterisks beside means for individual lines in bold type indicate values significantly different from wild-type (WT) by post analysis-of-variance (ANOVA) *t*-test. Asteriks beside the means for WT in bold type indicate values significantly different from both lines by post ANOVA contrast; *P ≤10%, **P ≤5%, ***P ≤1%. Click here for file

Additional file 7 **List of PCR primers used in the study.** Sequences of all forward and reverse PCR primers used in this study. Click here for file
