# Supplementary material for: Aspen pectate lyase PtxtPL1-27 mobilizes matrix polysaccharides from woody tissues and improves saccharification yield
Source: Biotechnol Biofuels. 2014 Jan 22;7:11. doi: 10.1186/1754-6834-7-11 (PMC3909318; doi:10.1186/1754-6834-7-11)
Supplement: Additional file 1 — Detection of PtxtPL1-27 transcript (EU379971.1) by northern blot. Northern blot analysis showing the size of PtxtPL1-27 transcript in the wild-type (WT) at 1.9 kb as compared to transcript size in four independent transgenic lines overexpressing PtxtPL1-27 ORF (without untranslated regions (UTRs)) under control of the CaMV 35S promoter at 1.4 kb. A polyubiquitin probe was used as a reference control. The RNA used in the northern blot analysis originated from developing xylem tissue. [file 1754-6834-7-11-S1.pptx]

## Slide 1
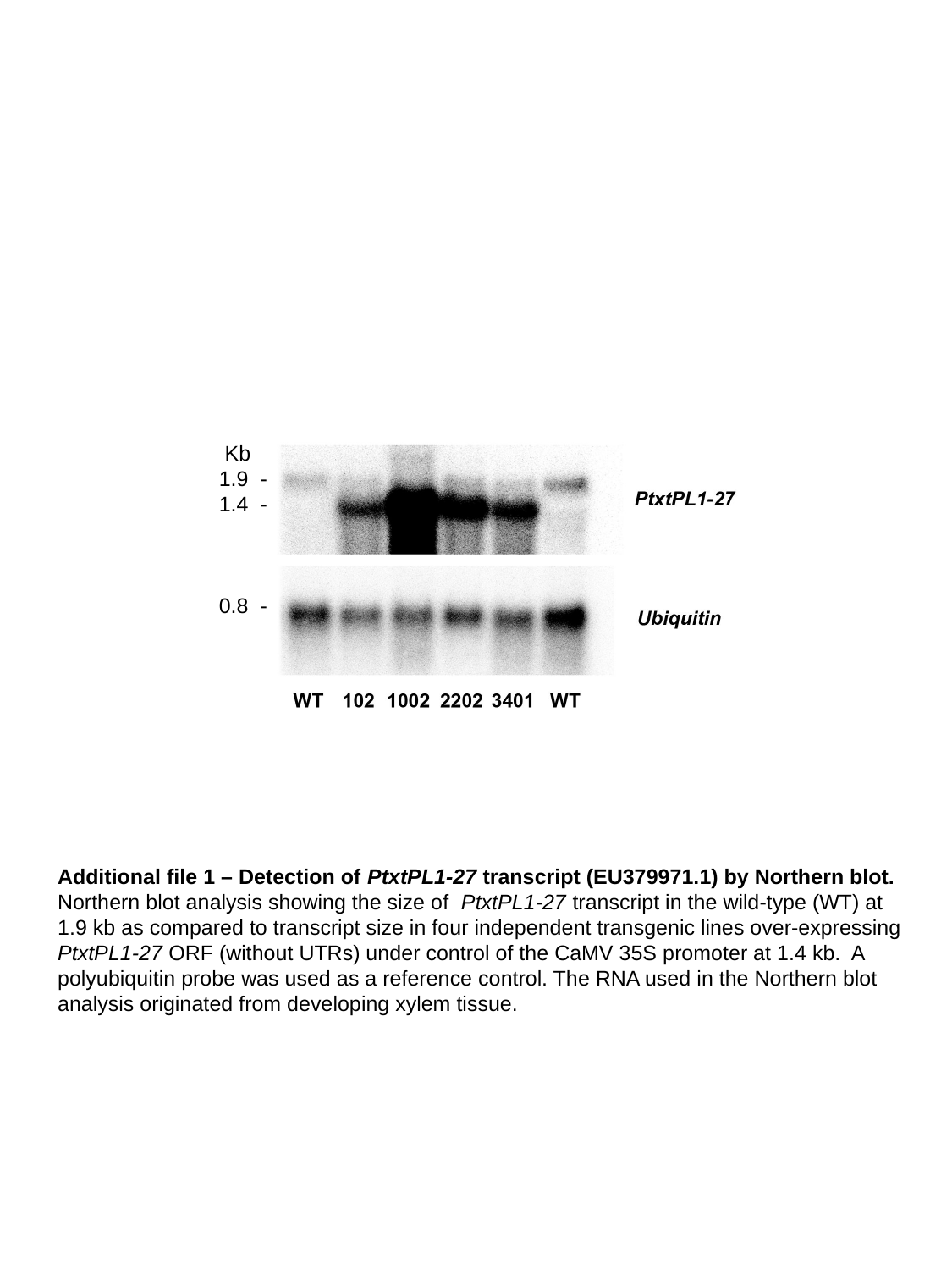

Kb
1.9 -
1.4 -
0.8 -
Additional file 1 – Detection of PtxtPL1-27 transcript (EU379971.1) by Northern blot.
Northern blot analysis showing the size of PtxtPL1-27 transcript in the wild-type (WT) at 1.9 kb as compared to transcript size in four independent transgenic lines over-expressing PtxtPL1-27 ORF (without UTRs) under control of the CaMV 35S promoter at 1.4 kb. A polyubiquitin probe was used as a reference control. The RNA used in the Northern blot analysis originated from developing xylem tissue.
