# Supplementary material for: Aspen pectate lyase PtxtPL1-27 mobilizes matrix polysaccharides from woody tissues and improves saccharification yield
Source: Biotechnol Biofuels. 2014 Jan 22;7:11. doi: 10.1186/1754-6834-7-11 (PMC3909318; doi:10.1186/1754-6834-7-11)
Supplement: Additional file 3 — SDS-PAGE showing the degree of purification of Δ 1-26 Ptxt PL1-27 expressed in E. coli. PtxtPL1-27 protein was expressed in E. coli using pTYB11 vector and partially purified by affinity chromatography. Proteins were separated by gel electrophoresis and stained by Coomassie. Lane 1: proteins extracted from E. coli expressing recombinant protein before induction. Lane 2: proteins extracted from E. coli expressing recombinant protein after induction by 0.3 mM isopropyl β-D-thiogalactoside (IPTG). The induced product can be seen at 98.8 kDa polypeptide representing PtxtPL1-27 fused to intein-chitin binding domain. Lane 3: partially purified recombinant protein is shown as 41.2 kDa band. The preparation is contaminated mainly by the 56.0 kDa polypeptide containing intein and chitin binding domain, which is the cleavage product of 98.8 kDa recombinant polypeptide. [file 1754-6834-7-11-S3.pptx]

## Slide 1
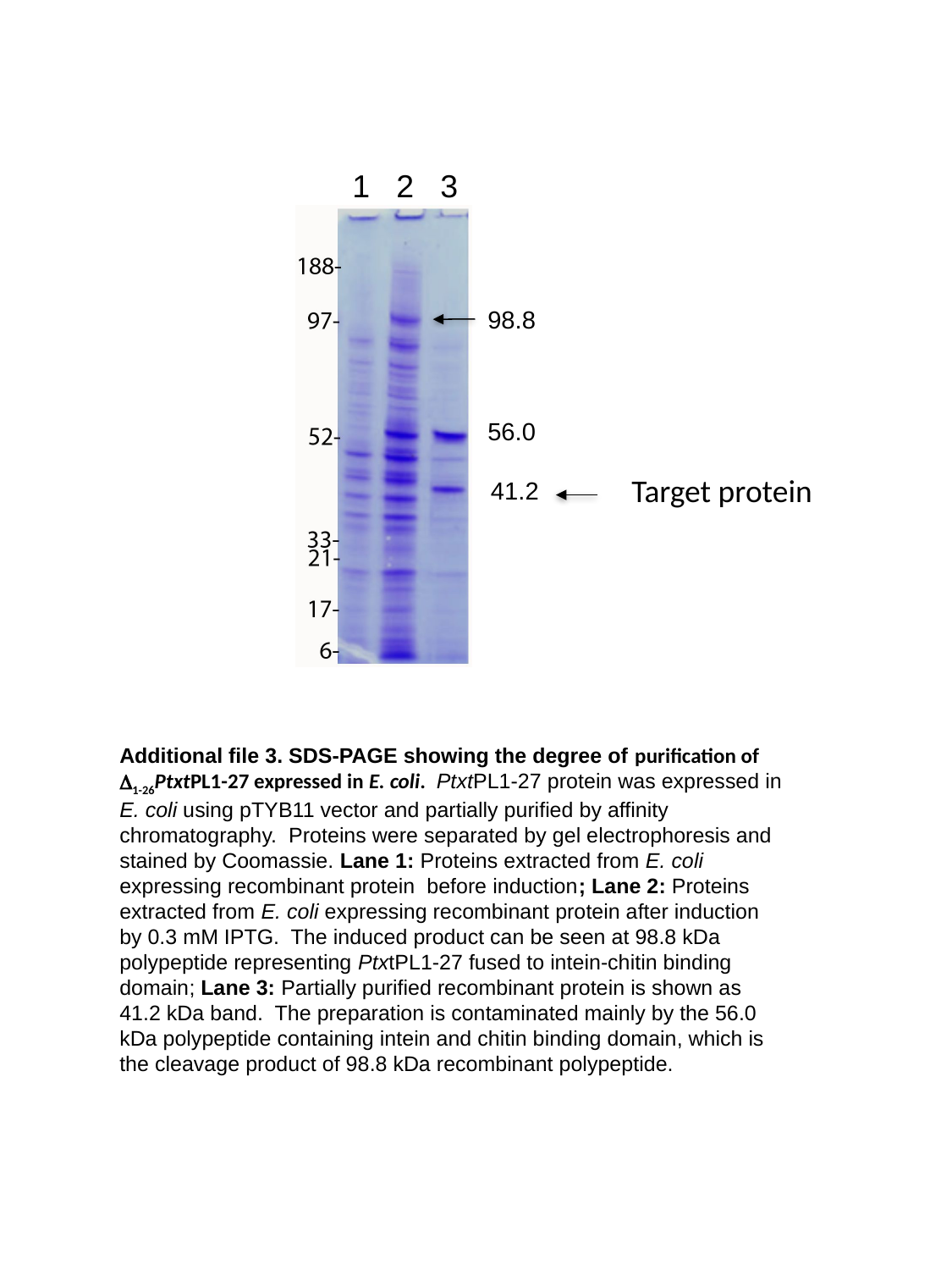

1 2 3
98.8
56.0
41.2
Target protein
Additional file 3. SDS-PAGE showing the degree of purification of D1-26PtxtPL1-27 expressed in E. coli. PtxtPL1-27 protein was expressed in E. coli using pTYB11 vector and partially purified by affinity chromatography. Proteins were separated by gel electrophoresis and stained by Coomassie. Lane 1: Proteins extracted from E. coli expressing recombinant protein before induction; Lane 2: Proteins extracted from E. coli expressing recombinant protein after induction by 0.3 mM IPTG. The induced product can be seen at 98.8 kDa polypeptide representing PtxtPL1-27 fused to intein-chitin binding domain; Lane 3: Partially purified recombinant protein is shown as 41.2 kDa band. The preparation is contaminated mainly by the 56.0 kDa polypeptide containing intein and chitin binding domain, which is the cleavage product of 98.8 kDa recombinant polypeptide.
