# Supplementary material for: Aspen pectate lyase PtxtPL1-27 mobilizes matrix polysaccharides from woody tissues and improves saccharification yield
Source: Biotechnol Biofuels. 2014 Jan 22;7:11. doi: 10.1186/1754-6834-7-11 (PMC3909318; doi:10.1186/1754-6834-7-11)
Supplement: Additional file 4 — Ectopic overexpression of PtxtPL1-27 inhibits plant growth. Data are relative stem increments and PtxtPL1-27 expression levels in elongating stem internodes of independent transgenic lines. Stem-length increments were determined as an increase in stem length during 64 days of growth in the greenhouse. Between two and eight plants per line were measured. Expression was determined by RT-q-PCR using 18S as a reference gene. [file 1754-6834-7-11-S4.pptx]

## Slide 1
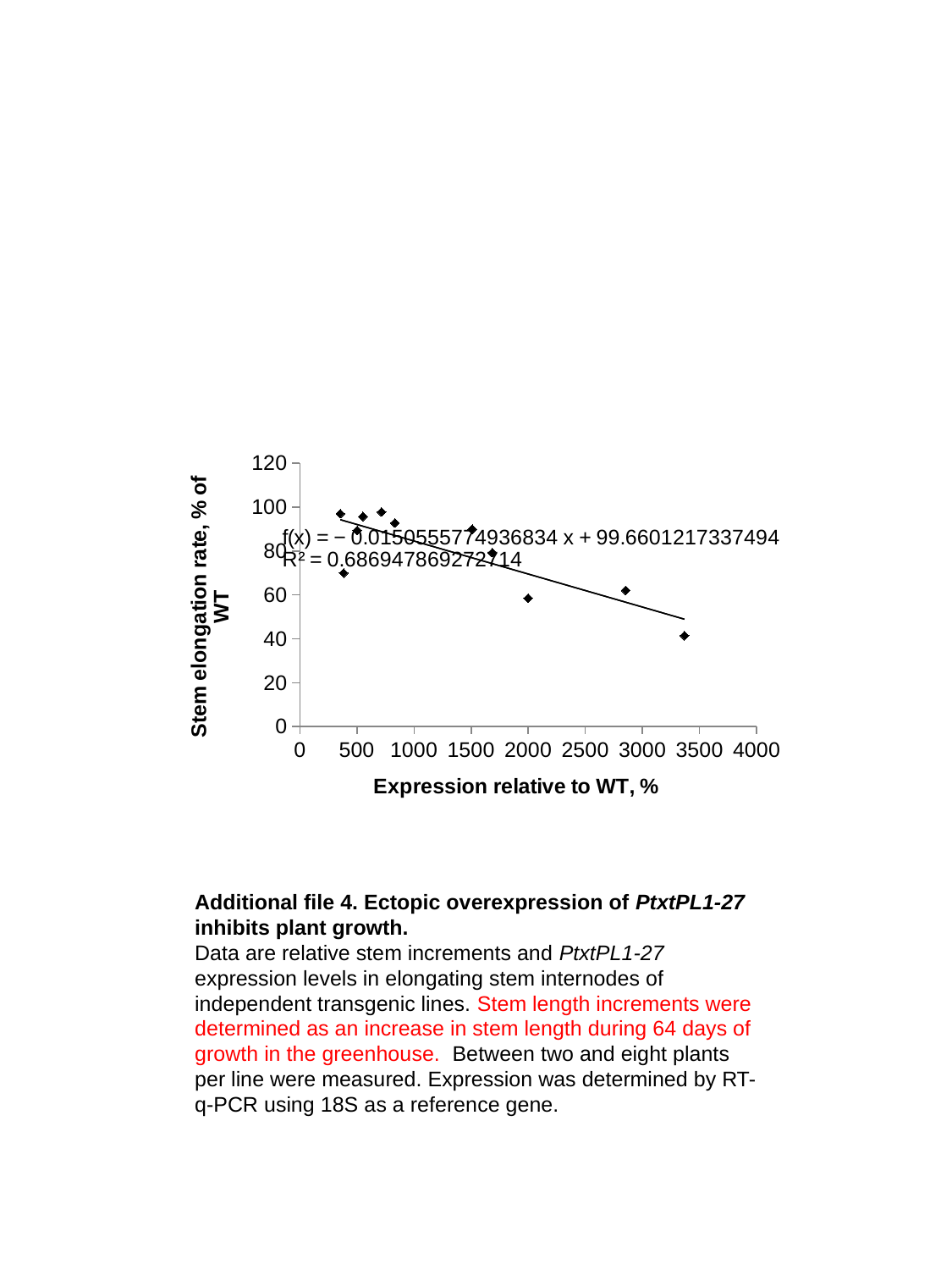

### Chart
| Category | |
|---|---|Additional file 4. Ectopic overexpression of PtxtPL1-27 inhibits plant growth.
Data are relative stem increments and PtxtPL1-27 expression levels in elongating stem internodes of independent transgenic lines. Stem length increments were determined as an increase in stem length during 64 days of growth in the greenhouse. Between two and eight plants per line were measured. Expression was determined by RT-q-PCR using 18S as a reference gene.
