# Supplementary material for: Aspen pectate lyase PtxtPL1-27 mobilizes matrix polysaccharides from woody tissues and improves saccharification yield
Source: Biotechnol Biofuels. 2014 Jan 22;7:11. doi: 10.1186/1754-6834-7-11 (PMC3909318; doi:10.1186/1754-6834-7-11)
Supplement: Additional file 6 — Monosaccharide composition of wood sequential extracts and the resulting pellets. Sugar composition of wood extracts; means ± standard error, n = 2 biological replicates. Asterisks beside means for individual lines in bold type indicate values significantly different from wild-type (WT) by post analysis-of-variance (ANOVA) t-test. Asteriks beside the means for WT in bold type indicate values significantly different from both lines by post ANOVA contrast; *P ≤10%, **P ≤5%, ***P ≤1%. [file 1754-6834-7-11-S6.docx]

**Additional file 6. Sugar composition of wood extracts;** means ± SE, N=2 biological replicates. Stars beside means for individual lines in bold type indicate values significantly different from WT by post-ANOVA t-test. Stars beside the means for WT in bold type indicate values significantly different from both lines by post-ANOVA contrast. * - P≤ 10%** - P ≤5% ; *** - P ≤1%.

|  | Sugar anhydrate composition (µg/mg extract) | | | | | | | | |
| --- | --- | --- | --- | --- | --- | --- | --- | --- | --- |
|  | Rha | Fuc | Ara | Xyl | Man | Gal | Glc | UA | |
| **a-amylase** | |  |  |  |  |  |  |  |  |
| WT | 12 ± 0 | 3 ± 0 | 23 ± 2 | 44 ± 1 | 27 ± 1 | 55 ± 2 | 25 ± 2 | **24 ± 2*** | |
| 1051 | 13 ± 0 | 3 ± 0 | 25 ± 1 | 47 ± 2 | 27 ± 2 | 59 ± 1 | 26 ± 2 | 32 ± 2 | |
| 1002 | **16 ± 1**** | 2 ± 0 | 28 ± 1 | 46 ± 3 | 28 ± 1 | 60 ± 2 | 30 ± 2 | **36 ± 3**** | |
| **EPG/PME** | |  |  |  |  |  |  |  | |
| WT | **4 ± 0***** | 0 | 7 ± 1 | **22 ± 2 **** | 21 ± 1 | **31 ± 2*** | **10 ± 0***** | **51 ± 5 **** | |
| 1051 | **5 ± 0 **** | 0 | 9 ± 1 | **32 ± 1 **** | 25 ± 1 | 35 ± 1 | **13 ± 1**** | 70 ± 4 | |
| 1002 | **6 ± 0 **** | 0 | 11 ± 1 | **32 ± 2 **** | 26 ± 1 | 37 ± 1 | **15 ± 1**** | **77 ± 4 **** | |
| **CaCO_3_** | |  |  |  |  |  |  |  | |
| WT | **8 ± 1 **** | 0 | 11 ± 1 | 40 ± 2 | 24 ± 1 | **45 ± 2 **** | **13 ± 1 *** | **61 ± 2 **** | |
| 1051 | **12 ± 1**** | 0 | 16 ± 2 | 49 ± 2 | 26 ± 0 | **39 ± 1 **** | 16 ± 2 | **96 ± 4 **** | |
| 1002 | 10 ± 1 | 0 | 12 ± 2 | 34 ± 2 | 24 ± 1 | **38 ± 1**** | **19 ± 1**** | **83 ± 6 **** | |
| **1M KOH** | |  |  |  |  |  |  |  | |
| WT | **11 ± 1**** | 0 | 6 ± 1 | **620 ± 12**** | 2 ± 0 | **12 ± 1*** | 6 ± 0 | **35 ± 3 **** | |
| 1051 | 14 ± 1 | 0 | 7 ± 0 | 678 ± 20 | 2 ± 0 | **9 ± 1**** | 6 ± 0 | 25 ± 2 | |
| 1002 | **15 ± 1**** | 0 | 6 ± 0 | **710 ± 10**** | 2 ± 0 | **10 ± 1** | 5 ± 0 | **24 ± 2 **** | |
| **4M KOH** | |  |  |  |  |  |  |  | |
| WT | **9 ± 0 **** | 2 ± 0 | 7 ± 0 | 555 ± 17 | 24 ± 1 | **13 ± 1**** | **25 ± 0***** | **31 ± 2***** | |
| 1051 | 11 ± 1 | 2 ± 0 | 7 ± 0 | 522 ± 14 | 27 ± 1 | **16 ± 1**** | **30 ± 0**** | **15 ± 0 **** | |
| 1002 | **13 ± 1**** | 3 ± 0 | 8 ± 1 | **625 ± 7**** | 29 ± 2 | **18 ± 0**** | **33 ± 0**** | **19 ± 2 **** | |
| **pellet** |  |  |  |  |  |  |  |  | |
| WT | 14 ± 1 | 0 | 6 ± 0 | **284 ± 10*** | **13 ± 1**** | **46 ± 3*** | **332 ± 9*** | **19 ± 2*** | |
| 1051 | 12 ± 1 | 0 | **4 ± 1*** | 307 ± 9 | **9 ± 1**** | 54 ± 5 | **292 ± 13*** | 24 ± 1 | |
| 1002 | **24 ± 3**** | 0 | 8 ± 1 | **317 ± 8*** | **8 ± 1**** | **65 ± 4**** | 307 ± 6 | **28 ± 2*** | |
|  |  |  |  |  |  |  |  |  | |
