# Supplementary material for: Aspen pectate lyase PtxtPL1-27 mobilizes matrix polysaccharides from woody tissues and improves saccharification yield
Source: Biotechnol Biofuels. 2014 Jan 22;7:11. doi: 10.1186/1754-6834-7-11 (PMC3909318; doi:10.1186/1754-6834-7-11)
Supplement: Additional file 7 — List of PCR primers used in the study. Sequences of all forward and reverse PCR primers used in this study. [file 1754-6834-7-11-S7.docx]

**Additional file 7. List of PCR primers used.**

| Forward primers | | Reverse primers | |
| --- | --- | --- | --- |
| name | primer | name | primer |
| MF1 | 5’GGTGGTTGCTCTTCCAACACTTCAAGACCCGATGT-3’ | MR1 | 5’-GGTGGTCTGCAGCTAGCACTGATGGCCCCT-3’ |
| PttPL1-27AFor | 5’-TTGTTGTTCGTTGGTGTCGTGGCTACTTC-3’ | PttPL1-27ARev | 5’-TCTGTTCCTTCTCTGCTTCTACATTCCTGGTAAG-3’ |
| PttPL1-27BFor | 5’-CAGTCAAATTTAAGTGGTCA-3’ | PttPL1-27BRev | 5’-CGAGTGACAGGCAGAAGTAGC-3’ |
| 18S F | 5’- TCAACTTTCGATGGTAGGATAGTG-3’ | 18S R | 5’-CCGTGTCAGGATTGGGTAATTT-3’ |
|  |  | Race1 | 5’-GAGACGGTGATGGCAGTAGAACCC-3’ |
| F1 | 5’-GCAGGGATCCAATGACAATGGCGGTGCCTC-3’ | R1 | 5’-TGAAGGATCCTAGCACTGATGGCCCCTGCG-3’ |
